# The longitudinal bidirectional association between sarcopenia and cognitive function in community-dwelling older adults: Findings from the China Health and Retirement Longitudinal Study

**DOI:** 10.7189/jogh.13.04182

**Published:** 2023-12-27

**Authors:** Jiajia Zhang, Xiuqin Jia, Yingying Li, Haibin Li, Qi Yang

**Affiliations:** 1 Department of Radiology, Beijing Chaoyang Hospital, Capital Medical University, Beijing, China; 2 Key Laboratory of Medical Engineering for Cardiovascular Disease, Ministry of Education, Beijing, China; 3 Department of Cardiac Surgery, Beijing Chaoyang Hospital, Capital Medical University, Beijing, China; 4 Heart Center & Beijing Key Laboratory of Hypertension, Beijing Chaoyang Hospital, Capital Medical University, Beijing, China; 5 Beijing Municipal Key Laboratory of Clinical Epidemiology, Beijing, China; 6 Beijing Laboratory for Cardiovascular Precision Medicine, Beijing, China

## Abstract

**Background:**

Although an association between sarcopenia and cognitive function has been demonstrated, the directional association remains unclear. The present study aimed to evaluate the longitudinal reciprocal relationship and identify the possible temporal sequence between sarcopenia and cognitive function in older Chinese adults.

**Methods:**

Data were collected from the China Health and Retirement Longitudinal Study (CHARLS) baseline survey in 2011 and the follow-up survey in 2015. Cognitive function was measured by episodic memory and executive function. Sarcopenia status (non-sarcopenia, possible sarcopenia and sarcopenia) was defined based on the Asian Working Group for Sarcopenia 2019 criteria. Linear regression analysis and ordinal logistic regression analysis were employed to investigate the relationship between baseline sarcopenia status and follow-up cognition, as well as the association of baseline cognition with follow-up sarcopenia status, respectively. A cross-lagged panel analysis was performed to simultaneously evaluate the bidirectional association and the strength of the temporal relationship.

**Results:**

Of 2689 participants, the median age was 65.0 years and 1249 (46.5%) were female. After adjusting for potential confounders and baseline measurements, baseline sarcopenia status was dose-dependently associated with subsequent cognitive scores (β = -0.45; *P* for trend = 0.001), and baseline cognitive scores (in tertiles) were also dose-dependently associated with subsequent sarcopenia status (odds ratio (OR) = 0.86; *P* for trend = 0.017). The cross-lagged panel analysis indicated that the standardised effect size of sarcopenia status on cognitive function (β = -0.09; *P* < 0.001) is larger relative to the effect of cognitive function on sarcopenia status (β = -0.05; *P* = 0.019).

**Conclusions:**

There is a longitudinal, bidirectional relationship between sarcopenia status and cognitive function in older Chinese adults. Sarcopenia is likely the driving force in these dynamic associations. These ﬁndings imply that interventions in either sarcopenia or cognitive decline may have the ability to generate reciprocal benefits over time. More research is warranted to conﬁrm these ﬁndings and to further elucidate underlying causal pathways.

Population aging is accelerating globally, increasing age-related diseases that inflict significant financial burdens on individuals, families, and society [1]. Cognitive decline is a natural part of aging, but a greater decline than expected can progress to mild cognitive impairment and eventually dementia [2,3]. The global prevalence of dementia is projected to double every 20 years, with an estimated 131.5 million affected individuals by 2050 [4]. Sarcopenia, characterised by progressive loss of skeletal muscle, muscle strength, and/or physical performance, is also prevalent in the aging population, with an estimated 9.9 to 40.4% of community-dwelling older adults affected globally [5]. It has been demonstrated that sarcopenia is strongly associated with multiple adverse health outcomes, such as functional decline, disability, falls, and mortality [6,7]. Consequently, identifying potentially modifiable factors of cognition and sarcopenia and implementing appropriate interventions are critical in reducing subsequent adverse events.

As common features of aging, sarcopenia and cognitive decline have been demonstrated to be associated [8-12], while these studies are limited due to cross-sectional designs. A few longitudinal unidirectional studies have also focused on this topic. Cohorts from China, Japan, Mexico and Malaysia have shown that sarcopenia is independently associated with cognitive decline or mild cognitive impairment among older adults [2,13-15]. Moreover, two recent systematic reviews and meta-analyses have also supported this temporal unidirectional relationship [16,17]. However, the longitudinal effect of cognitive function on sarcopenia remains poorly explored. A most recent prospective study from Brazilian older adults has reported a predictive role of cognitive impairment on later sarcopenia [18]. Evidence from a meta-analysis has also suggested that cognitive impairment is one of the risk factors for sarcopenia [19]. Other available longitudinal research has primarily evaluated the association between cognition and sarcopenia components, showing an effect of cognitive decline on later slower gait speed [20,21] and weaker handgrip strength [22]. Nevertheless, these longitudinal studies are unidirectional, only evaluating either the effect of sarcopenia on cognition or the effect of cognition on sarcopenia, failing to determine the directional association.

To our knowledge, no previous longitudinal study has simultaneously explored the bidirectional association between cognition and sarcopenia or determined a potential temporal sequence. However, certain indications from existing research may imply a reciprocal relationship between these two factors. For instance, while the evidence is unidirectional, the studies mentioned above suggest that sarcopenia is associated with poor cognition, and vice versa. Moreover, some prospective investigations have identified bidirectional relationships between cognition and specific sarcopenia components, such as gait speed [23] and handgrip strength [24]. A recent meta-analysis has suggested a possible mutual association between sarcopenia and cognitive impairment, drawing from previous unidirectional longitudinal studies that focused on individual sarcopenia components [16]. Additionally, sarcopenia and cognitive decline may share common pathophysiological mechanisms, including chronic inﬂammation and oxidative stress [25,26].

Overall, sarcopenia and cognitive function are likely associated with each other. The prior evidence solidly supported the role of sarcopenia in cognitive decline, but cognition’s effect on sarcopenia and the possible reciprocal relationship remained incompletely understood. Clarifying the possible directionality of events could help us understand key mechanisms and make interventional efforts to mitigate both cognitive decline and sarcopenia. Furthermore, increasing age-related diseases pose significant challenges, particularly in low- and middle-income countries, such as China [1,27,28]. To bridge this research gap, the present study aimed to investigate the possible bidirectional relationship between sarcopenia and cognitive function in a community-based prospective cohort of older Chinese adults.

## METHODS

### Study population

This cohort study used data from the China Health and Retirement Longitudinal Study (CHARLS). The CHARLS is an ongoing nationally representative longitudinal survey of Chinese adults aged 45 years and older. Detailed information about the CHARLS can be found in previous literature [29]. Briefly, the baseline survey was conducted in 2011 using a multistage stratified probability-proportionate-to-size sampling method, and a total of 17 708 individuals were recruited from 28 provinces across China. All participants were followed up every two or three years by using face-to-face computer-assisted personal interviews. Besides, the first three surveys included physical measurements, and walking speed measurements were only conducted among the participants aged 60 years or older. The protocol was approved by the Ethical Review Committee of Peking University (IRB00001052-11015). All participants provided written informed consent before the survey.

In the present study, we used data from the baseline survey (wave 1, year 2011) and the second follow-up survey (wave 3, year 2015). We excluded 15 019 participants due to: 1) aged less than 60 years old or having no information about age; 2) self-reported diagnosis of dementia or Parkinson disease or both; 3) having brain damage or mental retardation; 4) missing data on sarcopenia or cognition, leaving 2689 participants eligible for the cross-lagged panel analysis.

**Figure 1:**
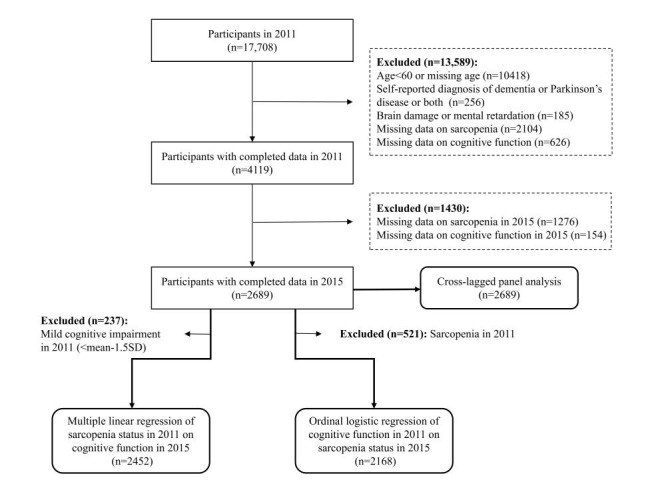
Study flowchart.

Besides, in the unidirectional longitudinal analyses, we further excluded adults (n = 237) with mild cognitive impairment ((MCI), cognitive scores < mean-1.5 standard deviation (SD)) [30] at baseline and those with sarcopenia at baseline (n = 521), respectively. Finally, a total of 2452 and 2168 initially unimpaired participants were included in linear regression analysis and ordinal logistic regression, respectively. The detailed selection of participants is illustrated in **Figure 1**.

### Measurement of cognitive function

In line with the previous studies [31,32], face-to-face assessments were used to measure cognitive function based on episodic memory and executive function. Episodic memory was assessed by immediate and delayed recall. After the interviewer randomly read ten Chinese words, participants were asked to recall them immediately and several minutes later, respectively. The episodic memory score was the sum of the recall points, ranging from 0 to 20. Executive function tests were mainly derived from the Telephone Interview for Cognitive Status, including orientation (week, month, day, season and year), calculation (serial subtraction of seven from 100 for five times) and visuospatial ability (drawing of the overlapping pentagons shown to them). The respondent scored one point for each correct item. The executive function score was the sum of these three tasks, with a range of 0 to 11 points. Global cognition was defined as the aggregated score of these two dimensions (score range, 0-31 points), with higher scores indicating better cognitive function.

### Measurement of sarcopenia

Sarcopenia was assessed based on the Asian Working Group for Sarcopenia 2019 (AWGS 2019) algorithm [33], including muscle strength, appendicular skeletal muscle mass (ASM), and physical performance. Possible sarcopenia is defined as low muscle strength or chair stand test ≥12 seconds (s) [33,34]. Sarcopenia is diagnosed when low ASM plus low muscle strength and/or low physical performance. Therefore, the participants were divided into three groups by sarcopenia severity (0 = non-sarcopenia; 1 = possible sarcopenia; 2 = sarcopenia) [13,35].

#### Muscle strength

Handgrip strength (kilogrammes (kg)) was measured in the dominant hand and non-dominant hand by squeezing YuejianTM WL-1000 dynamometer as hard as possible. The average of maximum available values with two hands was used. If the participant is unable to measure grip strength with either hand, we will use the value of the available hand. The cut-off points for low grip strength were <28 kg for men and <18 kg for women, respectively.

#### Appendicular skeletal muscle mass (ASM)

The ASM was estimated by a validated anthropometric equation in the Chinese population [36]: ASM = 0.193 × weight (kg) + 0.107 × height (cm) - 4.157 × sex - 0.037 × age (years) - 2.631.

If male, sex was set to 1, and female to 2. The strong agreement between the ASM equation model and dual x-ray absorptiometry (DXA) has been validated in previous studies [34,37]. The cut-off points for low muscle mass were based on the sex-speciﬁc lowest 20% of the height-adjusted muscle mass (ASM/height^2^) of the study population [13,38], with <6.86 kg/square metres (m^2^) in men and <5.08 kg/m^2^ in women at baseline, and <6.80 kg/m^2^ in men and <4.99 kg/m^2^ in women at follow-up.

#### Physical performance

Usual gait speed and 5-time chair stand test were performed to assess physical performance [35,38]. Gait speed (metre per second (m/s)) was evaluated by measuring the usual speed on a 2.5-m walking course. Participants were instructed to walk the 2.5-m course at their usual pace. This process involved walking from one end of the course to the other and then back again, with a stopwatch used to record the time taken. We calculated the average of the available values obtained from two trials. The chair stand test assessed the time (in seconds) required for participants to rise continuously from a 47-centimetres (cm) high chair five times, while keeping their arms folded across their chest. The cut-off point of low physical performance was gait speed <1.0 m/s or 5-time chair stand test ≥12 seconds [35,38]. Besides, individuals who tried but failed to complete either of these two tests were also considered as having low physical performance.

### Covariates

According to previous research [9,13,17,39-42], sociodemographic characteristics, lifestyle factors, and major clinical factors were selected as potential covariates. Sociodemographic characteristics included age, sex (female and male), marital status (married and others), residence (urban and rural), educational level (no formal education, primary school, middle school, and high school or above), and average household income (quartiles). Lifestyle factors included smoking and drinking status (yes or no), social isolation, nighttime sleep duration, and post-lunch napping duration. Major clinical factors included body mass index (BMI), malnutrition (yes or no), depressive symptoms (yes or no), restrictions on activities of daily living (ADL) (yes or no), history of 13 common diseases (hypertension, diabetes, cancer, lung disease, heart diseases, stroke, arthritis, dyslipidaemia, kidney disease, asthma, digestive disease, emotional and mental disorders, and liver disease), comorbidity index, and visual and hearing impairment (yes or no).

The social isolation index was calculated by three items [42]. Participants were assigned one point if were not married, had less than weekly contact (by phone, in person, or by e-mail) with children, or did not participate in any social activities during the last month. The total score of the isolation ranged from 0 to 3. Night time sleep duration was categorised into four groups (<5 hours (h), 5-6.9 hours, 7-8.9 hours, and ≥9 hours) [41]. Post-lunch napping duration was divided into three groups (<30 minutes (min), 30-90 min and ≥90 min) [41]. BMI was divided into four groups: underweight (<18.5 kg/m^2^), normal (18.5-23.9 kg/m^2^), overweight (24.0-27.9 kg/m^2^) and obese (≥28.0 kg/m^2^) [31]. Participants were regarded as malnutrition if they had BMI malnutrition (<18.5 kg/m^2^) or weight loss malnutrition (weight loss of >5 kg in the last year) [43]. Depressive symptoms were assessed using the 10-item version of the Center for Epidemiologic Studies Depression Scale and a score of ≥10 was considered as depressive symptoms [44]. Restrictions on ADL were defined as having difﬁculties in one or more of the ﬁve daily activities including bathing, dressing, eating, getting in/out of bed, and toileting [45]. Comorbidity was classified into three groups (0, 1 or ≥2) according to the number of 13 diseases.

### Statistical analyses

Continuous variables were summarised as means (standard deviations (SDs)) or medians (interquartile range (IQR)) and compared using unpaired *t* tests/one-way analysis of variance (ANOVA) analyses or Wilcoxon tests/Kruskal-Wallis tests. Categorical variables were presented with numbers (proportions) and compared by χ^2^ tests.

#### Longitudinal unidirectional analyses

Linear regression analysis was conducted to investigate the association between baseline sarcopenia status and follow-up cognitive scores (continuous) among the participants without baseline MCI (cognitive scores < mean - 1.5 SD) [30]. Then ordinal logistic regression was used to analyse the association between baseline cognitive scores (categorised into tertiles) and follow-up sarcopenia status after excluding individuals with sarcopenia at baseline. Regression coefficients (β) or odds ratios (ORs) and corresponding 95% confidence intervals (CIs) were documented from 4 models. Model 1 was a crude model. Model 2 was adjusted for age and sex. Model 3 was additionally adjusted for the other variables that were significantly imbalanced between the two groups by MCI (Table S1 in the **Online Supplementary Document**) or the three groups by sarcopenia (Table S2 in the **Online Supplementary Document**). Model 4 was further controlled for the baseline levels. Trend tests were assessed by treating sarcopenia and cognitive scores as ordinal variables. Multicollinearity was examined using the Variance Inflation Factor ((VIF) >4 indicates multicollinearity) [46].

#### Cross-lagged panel analysis

To examine the bidirectional association between sarcopenia and cognitive function simultaneously, a cross-lagged panel model (CLPM) was performed among the total participants (n = 2689). This analysis examined how the two factors influence each other over time after accounting for their baseline levels and evaluated the strength of the temporal relationship, which provided stronger evidence for the temporal sequence [40,47]. A fully-adjusted CLPM was built, adjusting for baseline sociodemographic characteristics (age, sex, marital status, residence and educational level), lifestyle factors (drinking and smoking status, social isolation and nighttime sleep and post-lunch napping duration) and major clinical factors (BMI category, malnutrition, depressive symptoms, restriction on ADL, comorbidity, and visual and hearing impairment). The CLPM was estimated using the means and variance-adjusted weighted least squares estimator (WLSMV), which is specifically used for ordinal variables in Mplus [48-50]. Missing data were handled using pairwise deletion as the default strategy [49]. The root mean square error of approximation (RMSEA) <0.06, comparative fit index (CFI) >0.90, and Tucker-Lewis Index (TLI) >0.90 indicate good model fit [51].

### Sensitivity analyses

We conducted several sensitivity analyses. First, given that demographic characteristics may be important confounders, we further performed subgroup analyses by age (60-69 or >69 years), sex (female or male), and education level (no formal education or primary school and above) repeating the two unidirectional analyses using model 4. We examined the interaction effects of subgroup variables with sarcopenia/cognition by adding a product interaction term to the model. Second, based on the 2689 participants, we performed latent class analysis (LCA) and latent profile analysis (LPA) with distal outcomes to explore underlying subtypes of sarcopenia status and cognitive function that might drive subsequent cognitive decline and sarcopenia, respectively. Initially, LCA and LPA were employed to empirically categorise sarcopenia and cognitive subtypes at baseline, respectively. LCA categorised participants based on low muscle strength, low muscle mass, and low physical performance (each factor had two levels: presence or absence). LPA was conducted using the two cognitive function assessments (episodic memory and executive function). Consistent with a prior study [52], model selection was based on various fit statistics, including the Akaike information criterion (AIC), Bayesian information criterion (BIC), sample-size-adjusted BIC (SSA-BIC), Lo-Mendell-Rubin likelihood ratio test (LMR), and classification accuracy (i.e. entropy). Subsequently, multiple linear regression was conducted to assess the association between baseline latent sarcopenia classes and follow-up cognition, and ordinal logistic regression was used to assess the association between baseline latent cognitive profiles and follow-up sarcopenia status. The two models were adjusted for the same covariates as the CLPM, and baseline level of outcome. We further performed LCA with continuous outcome (cognitive scores) to provide comparisons among the classes of sarcopenia on the follow-up cognition, applying the automatic DCON method in Mplus [53]. For the LPA with categorical outcome (sarcopenia status), we used the DCAT command [53] to examine differences in the follow-up sarcopenia status between cognitive profiles. Third, in the main analyses, average household income was not adjusted as 24.2% (n = 651) of participants had missing data on this variable, thereby we further adjusted for average household income in sensitivity analyses. Fourth, we controlled for the specific history of diseases instead of a comorbidity index in the cross-lagged analysis. However, the coefficients obtained were the same when controlling for the 13 diseases as when using the comorbidity index. Therefore, we presented a more parsimonious model that only included the comorbidity index.

All analyses were conducted using R version 4.2.1 and Mplus version 8.0 [49]. Two-sided *P* < 0.05 was considered statistically significant.

## RESULTS

The baseline characteristics are presented in **Table 1**. A national sample of 2689 participants were ultimately included in the analysis, of whom 46.5% were female and 80.7% were married. The median (IQR) age of the population was 65.0 (62.0-69.0) years. More than two-thirds of the respondents lived in rural areas (67.0%) and over half had no formal education (52.0%).

**Table 1:** Baseline characteristics of the study participants

Characteristics	Value (n = 2689)†
**Age, year, median (IQR)**	65.0 (62.0-69.0)
**Sex (female), n (%)**	1249 (46.5)
**Married, n (%)**	2169 (80.7)
**Residence, n (%)**	
Urban	888 (33.0)
Rural	1801 (67.0)
**Educational level, n (%)**	
No formal education	1398 (52.0)
Primary school	787 (29.3)
Middle school	357 (13.3)
High school or above	147 (5.5)
Average household income*, CNY, n (%)	
Quartile 1 (<800)	517 (25.4)
Quartile 2 (800-3118.3)	502 (24.6)
Quartile 3 (3118.3-8226.9)	510 (25.0)
Quartile 4 (8226.9-95000)	509 (25.0)
**Current drinker, n (%)**	880 (32.7)
**Current smoker, n (%)**	901 (33.5)
Social isolation*, median (IQR)	1.0 (0.0-1.0)
Nighttime sleep duration*, hours, n (%)	
<5.0	499 (18.7)
5.0-6.9	912 (34.1)
7.0-8.9	1035 (38.7)
≥9.0	227 (8.5)
Post-lunch napping duration*, minutes, n (%)	
<30	1433 (53.4)
30-90	831 (31.0)
≥90	419 (15.6)
**BMI category, n (%)**	
Underweight	215 (8.0)
Normal	1509 (56.1)
Overweight	704 (26.2)
Obese	261 (9.7)
Malnutrition*, n (%)	374 (13.9)
**Depressive symptoms, n (%)**	981 (36.5)
Restriction on ADL*, n (%)	409 (15.3)
**History of diseases, n (%)**	
Hypertension*	850 (31.7)
Diabetes*	184 (6.9)
Cancer*	18 (0.7)
Lung disease*	345 (12.9)
Heart diseases*	397 (14.8)
Stroke*	58 (2.2)
Arthritis*	930 (34.6)
Dyslipidaemia*	258 (9.8)
Kidney disease*	156 (5.8)
Asthma*	181 (6.8)
Digestive disease*	610 (22.8)
Emotional and mental disorders*	31 (1.2)
Liver disease*	74 (2.8)
**Comorbidity, n (%)**	
0	730 (27.1)
1	795 (29.6)
≥2	1164 (43.3)
Visual impairment*, n (%)	212 (7.9)
Hearing impairment*, n (%)	289 (10.8)
**Sarcopenia status**	
Non-sarcopenia	1315 (48.9)
Possible sarcopenia	853 (31.7)
Sarcopenia	521 (19.4)
**Cognitive score, mean ± SD**	14.1 ± 5.1

IQR – interquartile range, CNY – Chinese Yuan, BMI – body mass index, CESD-10 – Center for Epidemiologic Studies Depression, ADL – activities of daily living, SD – standard deviation

*Missing data: 651 for average household income, 50 for social isolation, 16 for nighttime sleep duration, 6 for post-lunch napping duration, 2 for malnutrition, 19 for restriction on ADL, 8 for hypertension, 20 for diabetes, 12 for cancer, 6 for lung disease, 13 for heart diseases, 4 for stroke, 3 for arthritis, 43 for dyslipidaemia, 18 for kidney disease, 10 for asthma, 8 for digestive disease, 10 for emotional and mental disorders, 15 for liver disease, 2 for visual impairment, 1 for hearing impairment.

†Values of variables may not sum to 100% due to rounding.

**Figure 2:**
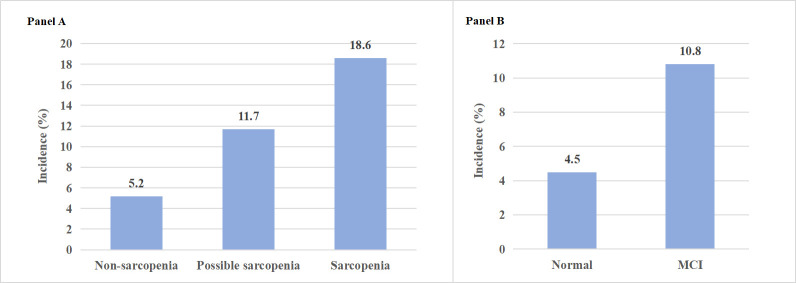
Incidence rates of new-onset MCI and sarcopenia across different baseline groups. **Panel A** shows the incidence rates of MCI by baseline sarcopenia status groups (non-sarcopenia, possible sarcopenia, and sarcopenia). **Panel B** shows the incidence rates of sarcopenia by baseline cognitive function groups (normal cognition and MCI). MCI – mild cognitive impairment.

In the longitudinal sample comprising 2452 participants, 237 individuals (9.7%) experienced new-onset MCI (cognitive scores < mean -1.5 SD) during the follow-up period. The incidence rates of MCI in the baseline non-sarcopenia, possible sarcopenia, and sarcopenia groups were 5.2, 11.7 and 18.6%, respectively, showing a significant difference (*P* < 0.001) (**Figure 2**, panel A). Furthermore, among the longitudinal cohort of 2168 individuals, 167 older adults (9.7%) developed new-onset sarcopenia during the follow-up. The incidence rates of sarcopenia in the baseline normal cognitive function and MCI groups were 4.5 and 10.8%, respectively, also displaying a significant difference (*P* < 0.001) (**Figure 2**, panel B).

### Influence of baseline sarcopenia on follow-up cognition

The linear regression analysis was conducted on 2452 participants without MCI at baseline. Baseline sarcopenia status was dose-dependently associated with follow-up cognitive scores in the crude model (β = -1.68, *P* for trend <0.001; **Table 2**). This association remained significant after adjusting for potential confounders (β = -1.13 and β = -0.64 in model 2 and model 3, respectively, all *P* for trend <0.001; **Table 2**). Moreover, the dose-response association was maintained after adjusting for baseline cognitive scores (β = -0.45, *P* for trend = 0.001, model 4; **Table 2**).

**Table 2:** Association between baseline sarcopenia and follow-up cognitive function*

Cognitive function	Non-sarcopenia (n = 1249)	Possible sarcopenia (n = 752)		Sarcopenia (n = 451)		β (95% CI)**	*P* for trend**
		**β (95% CI)**	***P*-value**	**β (95% CI)**	***P*-value**		
Model 1† (n = 2452)	0 (reference)	-2.26 (-2.74, -1.78)	<0.001	-3.12 (-3.69, -2.56)	<0.001	-1.68 (-1.95, -1.41)	<0.001
Model 2‡ (n = 2452)	0 (reference)	-1.74 (-2.20, -1.28)	<0.001	-1.98 (-2.55, -1.42)	<0.001	-1.13 (-1.40, -0.86)	<0.001
Model 3§ (n = 2351)‖	0 (reference)	-1.05 (-1.46, -0.64)	<0.001	-0.92 (-1.54, -0.29)	0.004	-0.64 (-0.93, -0.36)	<0.001
Model 4¶ (n = 2351)‖	0 (reference)	-0.81 (-1.18, -0.43)	<0.001	-0.55 (-1.13, 0.02)	0.062	-0.45 (-0.70, -0.19)	0.001

CI – confidence interval

*Linear regression analysis was used to model this association.

†Model 1 was a crude model.

‡Model 2 was adjusted for age and sex.

§Model 3 was additionally adjusted for marital status, residence, educational level, drinking status, social isolation, nighttime sleep duration, post-lunch napping duration, BMI category, malnutrition, depressive symptoms, dyslipidaemia, and comorbidity.

‖The model was performed with excluding missing data on covariates.

¶Model 4 was further additionally adjusted for baseline cognitive scores.

**β and *P* values for trend were calculated by treating baseline sarcopenia as ordinal variables (0 = non-sarcopenia; 1 = possible sarcopenia; 2 = sarcopenia) and β represents the change in cognitive score for each one-rank increase in baseline sarcopenia status.

### Influence of baseline cognition on follow-up sarcopenia

The ordinal logistic regression analysis of the 2168 participants without baseline sarcopenia showed that initial cognitive scores (in tertiles) were dose-dependently associated with subsequent sarcopenia status in the crude model (OR = 0.66, *P* for trend <0.001; **Table 3**). Controlling for covariates did not change this association (OR = 0.71, *P* for trend <0.001, model 2; OR = 0.83, *P* for trend = 0.004, model 3; **Table 3**). After further adjusting for the baseline sarcopenia, the dose-dependent association was still observed (OR = 0.86, *P* for trend *=* 0.017, model 4; **Table 3**).

**Table 3:** Association between baseline cognitive function and follow-up sarcopenia*

Sarcopenia status	Lowest tertile (n = 859)	Middle tertile (n = 658)		Highest tertile (n = 651)		OR (95% CI)**	*P* for trend**
		OR (95% CI)	*P*-value	OR (95% CI)	*P*-value		
Model 1† (n = 2168)	1.00 (reference)	0.66 (0.54-0.81)	<0.001	0.43 (0.35-0.53)	<0.001	0.66 (0.59-0.73)	<0.001
Model 2‡ (n = 2168)	1.00 (reference)	0.76 (0.61-0.93)	0.008	0.50 (0.40-0.62)	<0.001	0.71 (0.64-0.79)	<0.001
Model 3§ (n = 2057)‖	1.00 (reference)	0.89 (0.71-1.12)	0.391	0.68 (0.53-0.88)	0.004	0.83 (0.73-0.94)	0.004
Model 4¶ (n = 2057)‖	1.00 (reference)	0.90 (0.72-1.13)	0.374	0.73 (0.56-0.94)	0.015	0.86 (0.75-0.97)	0.017

OR – odds ratio, CI – confidence interval

*Ordinal logistic regression was used to model this association, and the OR is interpreted as the odds of being in a more severe sarcopenia status compared with the reference category.

†Model 1 was a crude model.

‡Model 2 was adjusted for age and sex.

§Model 3 was additionally adjusted for marital status, residence, educational level, drinking status, social isolation, nighttime sleep duration, BMI category, depressive symptoms, restriction on activities of daily living (ADL), hypertension, cancer, stroke, arthritis, and dyslipidaemia.

‖The model was performed with excluding missing data on covariates.

¶Model 4 was further additionally adjusted for baseline sarcopenia status.

**OR and *P* values for trend were calculated by treating baseline cognitive score as ordinal variables (0 = lowest tertile; 1 = middle tertile; 2 = highest tertile) and OR indicates the odds ratio of being in a more severe sarcopenia status for each one-rank increase in baseline cognitive scores (tertiles).

### Longitudinal bidirectional association between sarcopenia and cognition

**Figure 3:**
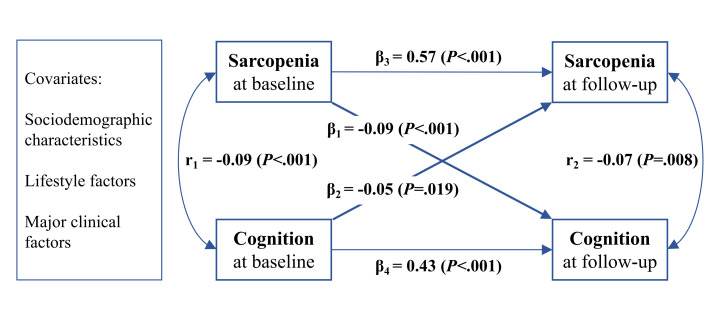
Cross-lagged panel model of sarcopenia and cognitive function. Sarcopenia was treated as ordinal variables (0 = non-sarcopenia; 1 = possible sarcopenia; 2 = sarcopenia). The cross-lagged panel model was estimated with the means and variance-adjusted WLSMV that was specifically developed for ordinal variables in Mplus. Standardised coefficients were reported. Single-headed arrows represented regression paths. Double-headed arrows represented correlations. The model was controlled for baseline sociodemographic characteristics (age, sex, marital status, residence and educational level), lifestyle factors (drinking and smoking status, social isolation and nighttime sleep and post-lunch napping duration) and major clinical factors (BMI category, malnutrition, depressive symptoms, restriction on ADL, comorbidity, and visual and hearing impairment). WLSMV – weighted least squares estimator, BMI – body mass index, ADL – activities of daily living.

A two-wave cross-lagged panel model was further built to simultaneously explore the bidirectionality. The final model was saturated (RMSEA = 0.00, CFI = 1.00 and TLI = 1.00). **Figure 3** shows the standardised coefficients. Baseline sarcopenia status was still significantly associated with subsequent cognitive function (β_1_ = -0.09; *P* < 0.001), although accounting for the strong intercorrelation between baseline and follow-up cognition (β_4_ = 0.43; *P* < 0.001). There was a similar association between baseline cognition and subsequent sarcopenia status (β_2_ = -0.05, *P* = 0.019; β_3_ = 0.57, *P* < 0.001).

### Sensitivity analyses

**Figure 4:**
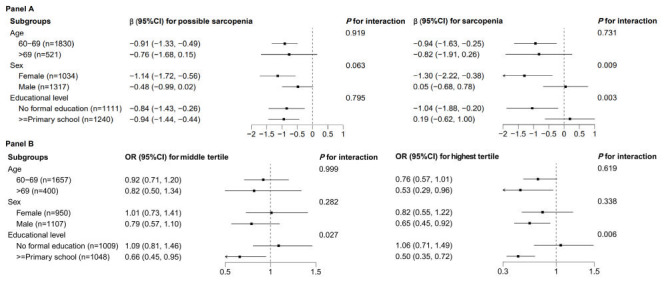
Subgroup analyses according to age, sex, and education in the two unidirectional longitudinal analyses. **Panel A**, the difference in cognitive function for possible sarcopenia group and sarcopenia group compared with the non-sarcopenia group across subgroups in the fully adjusted models (n = 2351). **Panel B**, odds ratios in more severe sarcopenia status for middle tertile of cognitive scores and highest tertile of cognitive scores compared with the lowest tertile of cognitive scores across subgroups in the fully adjusted models (n = 2057).

In the subgroup analyses, we observed significant interactions for sex and education in the longitudinal effect of sarcopenia status on cognitive function, but age did not show this effect (**Figure 4**, panel A). Specifically, the mean differences between sarcopenia and non-sarcopenia groups were -1.30 (95% CI = -2.22, -0.38) for females and 0.05 (95% CI = -0.68, 0.78) for males (*P* for interaction = 0.009). Similarly, the mean differences were -1.04 (95% CI = -1.88, -0.20) for individuals with no formal education and 0.19 (95% CI = -0.62, 1.00) for those with a primary school education or higher (*P* for interaction = 0.003). Moreover, the level of education also influenced the association between baseline cognitive scores and follow-up sarcopenia status, with more pronounced effects seen in individuals with primary education or above (*P* for interaction = 0.027 for middle tertile of cognitive scores and *P* for interaction = 0.006 for highest tertile of cognitive scores) (**Figure 4**, panel B). Comparable results to the main analyses were observed according to age and sex (all *P* for interaction >0.05).

**Figure 5:**
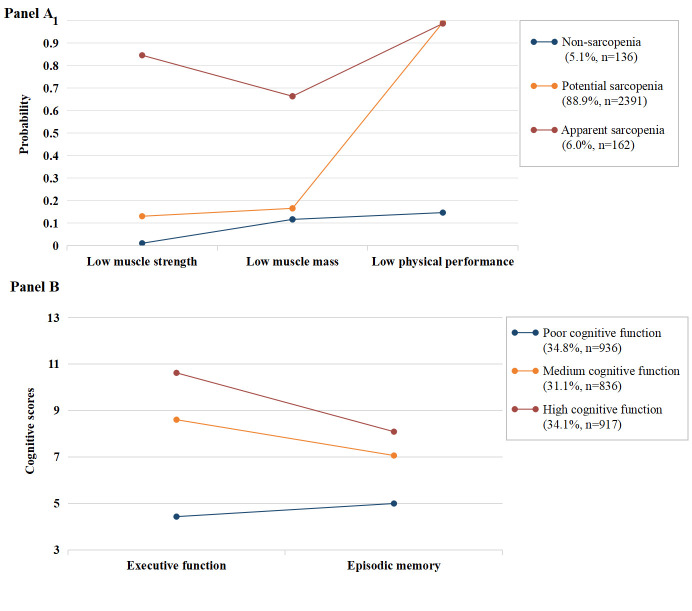
Latent sarcopenia status classes (**Panel A**) and latent cognitive profiles (**Panel B**) of the 2689 participants at baseline.

Table S3 in the **Online Supplementary Document** provides the fit indices of LCA and LPA at baseline. Two 3-profile models were considered optimal (**Figure 5**). For the LCA, although 2-class exhibited slightly lower AIC, BIC and SSABIC values, the entropy was very small (entropy = 0.374), which indicated more classification errors. Along with the consideration of theoretical meaning of solutions, we selected the 3-class model: non-sarcopenia, potential sarcopenia, and apparent sarcopenia (**Figure 5**, panel A). For LPA, the 3-profile solution was chosen, as it had lower AIC, BIC, and SSABIC values, larger entropy, and significant LRT-LMR value, in comparison to 2-profile solution. Although the 4-profile and 5-profile solutions had slightly lower AIC, BIC, SSABIC values than 3-profile solution, their entropy values were lower, and LRT-LMR value of the 5-profile was non-significant. As a note, the membership in one class of the 4-profile comprised less than 5% (n = 134) of the sample, which may reduce the accuracy [54]. Therefore, we retained the 3-profile model (**Figure 5**, panel B): the high cognitive function (with higher scores for both assessments), medium cognitive function (with moderate scores for both assessments), and poor cognitive function (with lowest scores for both assessments).

**Table 4** and **Table 5** showed the results of latent sarcopenia classes predicting follow-up cognition and latent cognitive profiles predicting follow-up sarcopenia status, which supported the longitudinal bidirectional association between sarcopenia and cognition. After multivariable adjustment, significant dose-response associations were found. Moreover, when compared with individuals in the status of non-sarcopenia, those with apparent sarcopenia status had poorer cognition function (β = -1.06, *P* = 0.033, model 4). Compared with older adults with poor cognitive function, those with medium cognitive function and high cognitive function displayed reduced risks of having more severe sarcopenia status (OR = 0.80, *P* = 0.038 and OR = 0.73, *P* = 0.009, respectively, model 4). A significant bidirectional relationship was also observed when using auxiliary variable (option DCON and DCAT in Mplus) to model LCA and LPA with distal outcomes (Table S4 in the **Online Supplementary Document**).

**Table 4:** Association between baseline latent classes of sarcopenia status and follow-up cognitive function*

Cognitive function	Non-sarcopenia (n = 136)	Potential sarcopenia (n = 2391)		Apparent sarcopenia (n = 162)		β (95% CI)**	*P* for trend**
		β (95% CI)	*P*-value	β (95% CI)	*P*-value		
Model 1† (n = 2689)	0 (reference)	-2.58 (-3.56, -1.60)	<0.001	-6.06 (-7.35, -4.77)	<0.001	-3.06 (-3.71, -2.42)	<0.001
Model 2‡ (n = 2689)	0 (reference)	-0.76 (-1.56, 0.04)	0.061	-1.83 (-2.91, -0.76)	0.001	-0.93 (-1.46, -0.39)	0.001
Model 3§ (n = 2598)‖	0 (reference)	-0.59 (-1.38, 0.20)	0.145	-1.59 (-2.69, -0.49)	0.005	-0.80 (-1.35, -0.25)	0.005
Model 4¶ (n = 2598)‖	0 (reference)	-0.40 (-1.11, 0.30)	0.265	-1.06 (-2.04, -0.08)	0.033	-0.53 (-1.02, -0.04)	0.033

CI – confidence interval.

*Linear regression analysis was used to model this association.

†Model 1 was a crude model.

‡Model 2 was adjusted for sociodemographic characteristics (age, sex, marital status, residence, and educational level).

§Model 3 was additionally adjusted for lifestyle factors (drinking and smoking status, social isolation, and nighttime sleep and post-lunch napping duration), and major clinical factors (BMI category, malnutrition, depressive symptoms, restriction on ADL, comorbidity, and visual and hearing impairment).

‖The model was performed with excluding missing data on covariates.

¶Model 4 was further additionally adjusted for baseline cognitive scores.

**β and *P* values for trend were calculated by treating baseline latent sarcopenia classes as ordinal variables (0 = non-sarcopenia; 1 = potential sarcopenia; 2 = apparent sarcopenia) and β represents the change in cognitive score for each one-rank increase in baseline latent sarcopenia classes.

**Table 5:** Association between baseline latent cognitive profiles and follow-up sarcopenia status*

Sarcopenia status	Poor cognitive function (n = 936)	Medium cognitive function (n = 836)		High cognitive function (n = 917)		OR (95% CI)**	*P* for trend**
		OR (95% CI)	*P*-value	OR (95% CI)	*P*-value		
Model 1† (n = 2689)	0 (reference)	0.52 (0.43-0.62)	<0.001	0.38 (0.32-0.45)	<0.001	0.61 (0.56-0.67)	<0.001
Model 2‡ (n = 2689)	0 (reference)	0.68 (0.56-0.82)	<0.001	0.58 (0.47-0.72)	<0.001	0.76 (0.69-0.85)	<0.001
Model 3§ (n = 2598)‖	0 (reference)	0.71 (0.58-0.88)	0.001	0.65 (0.52-0.82)	<0.001	0.81 (0.72-0.90)	<0.001
Model 4¶ (n = 2598)‖	0 (reference)	0.80 (0.64-0.99)	0.038	0.73 (0.57-0.92)	0.009	0.85 (0.76-0.96)	0.009

OR – odds ratio, CI – confidence interval

*Ordinal logistic regression was used to model this association, and the OR is interpreted as the odds of being in a more severe sarcopenia status compared with the reference category.

†Model 1 was a crude model.

‡Model 2 was adjusted for sociodemographic characteristics (age, sex, marital status, residence, and educational level).

§Model 3 was additionally adjusted for lifestyle factors (drinking and smoking status, social isolation, and nighttime sleep and post-lunch napping duration), and major clinical factors (BMI category, malnutrition, depressive symptoms, restriction on ADL, comorbidity, and visual and hearing impairment).

‖The model was performed with excluding missing data on covariates.

¶Model 4 was further additionally adjusted for baseline sarcopenia status.

**OR and *P* values for trend were calculated by treating baseline cognitive score as ordinal variables (0 = poor cognitive function; 1 = medium cognitive function; 2 = high cognitive function) and OR indicates the odds ratio of being in a more severe sarcopenia status for each one-rank increase in baseline latent cognitive profiles.

After further controlling for the average household income, the longitudinal dose-response relationship between sarcopenia and cognitive function was also found (β = -0.30, *P* for trend = 0.049, Table S5 in the **Online Supplementary Document**), but this dose-response association of baseline cognitive scores with follow-up sarcopenia status became marginally significant (OR = 0.88, *P* for trend *=* 0.087, Table S6 in the **Online Supplementary Document**). The bidirectional association observed in the main analysis did not change after additional adjustment for average household income (Figure S1 in the **Online Supplementary Document**).

## DISCUSSION

In a population-based prospective cohort of community-dwelling older Chinese adults aged 60 years or older, we observed a longitudinal, bidirectional association between sarcopenia status and cognitive function. The standardised cross-lagged path coefficient suggested that the direction from baseline sarcopenia status to subsequent cognitive function may be relatively stronger than the one from baseline cognition to subsequent sarcopenia.

Although previous cross-sectional studies have indicated an association of sarcopenia with cognition function and vice versa, they are limited to conclusions regarding the directionality [9,12]. The present study extends the cross-sectional association by examining the temporal bidirectional association. Consistent with prior research [2,13-15], we found a unidirectional longitudinal effect of initial sarcopenia on subsequent poor cognitive function. Two studies conducted among 496 community-dwelling Mexican adults (aged ≥50) [2] and 131 Japanese community-dwelling older adults (aged ≥65) [15], respectively, showed that sarcopenia predicted subsequent cognitive decline during follow-up periods of eight years and one year. Compared with the two longitudinal studies, our analysis was based on a larger sample size, enabling a more powerful statistical assessment. Recently, a study reported a unidirectional association between sarcopenia and mild cognitive impairment during a 3-year follow-up period [13]. The previous one and our study were both conducted in populations aged 60 and above from the CHARLS, and the sample sizes were relatively similar (previous study: n = 2982; the current study: n = 2689). However, our study extended the findings of the previous research. Specifically, the earlier study solely explored the impact of sarcopenia on subsequent cognitive impairment and did not examine the reverse pathway. In contrast, our study not only supported the result of the previous study but also identified a bidirectional relationship. However, previous prospective studies are lacking on the unidirectional relationship between cognitive function and subsequent sarcopenia. Only a recent longitudinal study reported an effect of cognitive impairment on the later development of sarcopenia in a population of community-dwelling older adults, which strengthens our findings [18]. Other available literature only revealed an effect of poorer cognition on follow-up slower gait speed [20,21] or less muscle strength [22]. Continued research is needed to test how cognition is associated with subsequent sarcopenia status, which may have important implications for clinicians.

In the trend test, a clear dose-dependent relationship between baseline sarcopenia status and subsequent cognitive function was observed. However, after adjusting for baseline cognition, the association between the baseline sarcopenia group and follow-up cognition became marginally significant. Interestingly, the stratified results revealed that the association was not statistically significant in males and those with higher education, which may potentially explain the marginal significance in the main analysis. Recent research on 286 older adults in South Korea has also highlighted sex differences, with sarcopenia exhibiting a significant association with cognitive impairment in older females but not in males [55]. These distinctions may be attributed to variances in gender-specific physiology [55]. Furthermore, participants with a higher level of education who had sarcopenia may have been more inclined to seek medical attention and receive care, potentially contributing to this marginal significance. Notably, the relationship between higher baseline cognitive scores and subsequently less severe sarcopenia status was only observed among participants with higher levels of education. Similarly, highly educated individuals may be more likely to manage their sarcopenia status at an early stage, potentially leading to a more pronounced beneficial effect on subsequent sarcopenia status.

Furthermore, we added a cross-lagged panel model to simultaneously examine the bidirectional association, finding that sarcopenia and cognitive function were reciprocally linked, and the greater sarcopenia could more likely be a cause than a result of poorer cognitive function. Although to date no such study to determine the interlinkage, some clues obtained from currently available studies might approve our results. For instance, a few previous studies have found a reciprocal relationship between cognitive function and sarcopenia’s components [23,24,56,57], with one [56] indicating that slower gait speed may be the dominating factor of the gait-cognition association. Moreover, a recent systematic review has also mentioned that the association of sarcopenia with cognitive impairment may be bidirectional [16], and our study contributes new evidence to support this bidirectionality. Gaining insights into the interplay and potential temporal sequence between cognitive decline and sarcopenia holds significant implications, not only for the early detection of these conditions but also for advancing the understanding of the underlying causal pathways.

It has been revealed that age-related cognitive decline and sarcopenia might share common mechanisms, which could help explain the findings. One mechanism is the role of chronic inﬂammation. High levels of pro-inflammatory factors interleukin-6 (IL-6), tumor necrosis factor-α (TNF-α), and C-reactive protein (CRP) may induce sarcopenia by affecting protein balance synthesis [2,58], and dysregulation of the inﬂammation may also be involved in the pathophysiological mechanisms of MCI [17,25]. Another possible mechanism is that excessive oxidative stress disrupts the balances of protein breakdown, protein synthesis, and apoptosis, subsequently leading to muscle atrophy and ﬁber loss [59], and that accumulative products of free radical damage are also presented in the central nervous system and peripheral tissues of patients with Alzheimer disease or MCI [26].

### Strengths and limitations

The present study has some strengths. Our work replicates previous findings on the longitudinal association between sarcopenia and cognitive function and extends it from unidirectional to bidirectional. To our knowledge, this study is an initial attempt to examine the reciprocal relationship of the two factors among the older population, which can identify the factor that contributes more to this association and help explain pathophysiological mechanisms. Moreover, this study utilised a nationally representative prospective cohort and controlled for various potential confounders, allowing for a relative reduction in biased estimates, and allowing for generalisation of the present findings to older Chinese general population.

Several limitations also need to be considered. First, this study was observational, which may result in biased relationships due to confounders. To address it, we considered a variety of covariates based on prior knowledge. Notably, given the potential mediating role of physical activity reported in previous literature [17], physical activity was not controlled. Second, this work was conducted among community-dwelling older adults, and our findings might not be generalisable to older adults in nursing homes or inpatients. More studies are necessary to fill this gap. Third, information on sarcopenia in the CHARLS is only available for the first three time points (2011, 2013 and 2015). Therefore, the follow-up period for this study was limited to four years. Similarly, this retrospective analysis used data collected approximately 8-12 years ago, which also constitutes a limitation of this study. Thus, research with relatively longer follow-up intervals applying the latest data are needed to confirm the current findings. Fourth, although the cross-lagged panel models allow for an examination of directional effects, causal inference, and biological mechanisms cannot be identified. Further studies are warranted to address this limitation. Finally, the present work only focused on cognitive function and sarcopenia rather than sarcopenia components. Therefore, the bi-directionality between individual sarcopenia components and cognitive function and which component contributes most to this association need to be further explored.

## CONCLUSIONS

In conclusion, the findings provide new evidence indicating a longitudinal bidirectional association between sarcopenia status and cognitive function in older Chinese adults. The observed bidirectionality implies that interventions in either sarcopenia or cognitive decline may have the ability to generate reciprocal benefits over time, potentially helping healthy aging. Moreover, the clear dose-dependent relationship and stronger cross-lagged coefficient suggest that sarcopenia might be the driving factor of the dynamic associations. Future research is needed to confirm these findings and elucidate the underlying mechanisms.

## Additional material


Online Supplementary Document
