# Supplementary material for: The longitudinal bidirectional association between sarcopenia and cognitive function in community-dwelling older adults: Findings from the China Health and Retirement Longitudinal Study
Source: J Glob Health. 2023 Dec 27;13:04182. doi: 10.7189/jogh.13.04182 (PMC10751559; doi:10.7189/jogh.13.04182)
Supplement: Online Supplementary Document [file jogh-13-04182-s001.pdf]

The longitudinal bidirectional association between sarcopenia and cognitive function in community-dwelling older adults: findings from the China Health and Retirement Longitudinal Study

Table S1. Baseline characteristics of the study participants without baseline mild cognitive impairment (n=2452)

Table S2. Baseline characteristics of the study participants without baseline sarcopenia (n=2168)

Table S3. Model fit and selection criteria for LCA and LPA at baseline

Table S4. Results of LCA and LPA with distal outcomes

Table S5. Association between baseline sarcopenia and follow-up cognitive function after additional adjustment for average household income[a]

Table S6. Association between baseline cognitive function and follow-up sarcopenia after additional adjustment for average household income[a]

Figure S1. Cross-lagged panel analysis of sarcopenia and cognitive function after additional adjustment for average household income

Table S1. Baseline characteristics of the study participants without baseline mild cognitive impairment (n=2452)

Characteristics	Without new-onset MCI (n=2215)	With new-onset MCI (n=237)	*P*-value
Age, year, median (IQR)	65.0 (62.0, 69.0)	69.0 (64.0, 74.0)	**<0.001**
Sex (female), n (%)	924 (41.7)	156 (65.8)	**<0.001**
Married, n (%)	1839 (83.0)	163 (68.8)	**<0.001**
Residence, n (%)			**<0.001**
Urban	758 (35.4)	53 (22.4)	
Rural	1430 (64.6)	184 (77.6)	
Educational level, n (%)			**<0.001**
No formal education	961 (43.4)	217 (91.6)	
Primary school	756 (34.1)	18 (7.6)	
Middle school	351 (15.8)	2 (0.8)	
High school or above	147 (6.6)	0 (0.0)	
Average household income[a], CNY, n (%)			**<0.001**
Quartile 1 (<800)	383 (22.9)	69 (37.5)	
Quartile 2 (800-3118.3)	391 (23.3)	51 (27.7)	
Quartile 3 (3118.3-8226.9)	430 (25.7)	43 (23.4)	
Quartile 4 (8226.9-95000)	472 (28.2)	21 (11.4)	
Current drinker, n (%)	780 (35.2)	55 (23.2)	**<0.001**

Current smoker, n (%)	778 (35.1)	63 (26.6)	**0.01**
Social isolation[a], median (IQR)	1.0 (0, 1.0)	1.0 (1.0, 1.0)	**<0.001**
Nighttime sleep duration[a], hours, n (%)			**0.005**
<5.0	370 (16.8)	58 (24.7)	
5.0-6.9	785 (35.6)	71 (30.2)	
7.0-8.9	876 (39.8)	81 (34.5)	
≥9.0	172 (7.8)	25 (10.6)	
Post-lunch napping duration[a], minutes, n (%)			**0.001**
<30	1142 (51.7)	152 (64.7)	
30-90	713 (32.2)	58 (24.7)	
≥90	356 (16.1)	25 (10.6)	
BMI (kg/m^2), median (IQR)	22.9 (20.6, 25.3)	21.6 (19.8, 24.1)	**<0.001**
BMI category, n (%)			**0.001**
Underweight	157 (7.1)	28 (11.8)	
Normal	1217 (54.9)	147 (62.0)	
Overweight	621 (28.0)	46 (19.4)	
Obese	220 (9.9)	16 (6.8)	
Malnutrition[a], n (%)	284 (12.8)	43 (18.1)	**0.029**
CESD-10 scores, median (IQR)	7.0 (3.0, 12.0)	9.0 (5.0, 14.0)	**<0.001**
Depressive symptoms, n (%)	755 (34.1)	111 (46.8)	**<0.001**

Restriction on ADL[a], n (%)[a]	306 (13.9)	41 (17.4)	0.179
History of diseases, n (%)			
Hypertension[a]	708 (32.1)	72 (30.4)	0.652
Diabetes[a]	159 (7.2)	11 (4.7)	0.186
Cancer[a]	17 (0.8)	0 (0.0)	0.344
Lung disease[a]	290 (13.1)	27 (11.4)	0.513
Heart diseases[a]	340 (15.4)	25 (10.5)	0.057
Stroke[a]	43 (1.9)	3 (1.3)	0.632
Arthritis[a]	760 (34.4)	83 (35.0)	0.895
Dyslipidemia[a]	229 (10.5)	14 (6.0)	**0.039**
Kidney disease[a]	136 (6.2)	12 (5.1)	0.612
Asthma[a]	153 (6.9)	16 (6.8)	>0.999
Digestive disease[a]	514 (23.3)	44 (18.6)	0.126
Emotional and mental disorders[a]	25 (1.1)	3 (1.3)	>0.999
Liver disease[a]	61 (2.8)	9 (3.8)	0.479
Comorbidity, n (%)			**0.032**
0	589 (26.6)	71 (30.0)	
1	636 (28.7)	81 (34.2)	
≥2	990 (44.7)	85 (35.9)	
Visual impairment[a], n (%)	161 (7.3)	23 (9.7)	0.222

Hearing impairment[a], n (%)	212 (9.6)	32 (13.5)	0.071
Sarcopenia status			**<0.001**
Non-sarcopenia	1184 (53.5)	65 (27.4)	
Possible sarcopenia	664 (30.0)	88 (37.1)	
Sarcopenia	367 (16.6)	84 (35.4)	

Notes: Statistical significance was assessed by unpaired t-test, Wilcoxon test, or a chi-squared test.

CNY, Chinese Yuan; BMI, body mass index; CESD-10, Center for Epidemiologic Studies Depression; ADL, activities of daily living.

Values of variables may not sum to 100% due to rounding.

P-values in bold indicate <0.05.

[a]Missing data: 592 for average household income, 47 for social isolation, 14 for nighttime sleep duration, 6 for post-lunch napping duration, 1 for malnutrition, 18 for restriction on ADL, 6 for hypertension, 18 for diabetes, 11 for cancer, 6 for lung disease, 11 for heart diseases, 3 for stroke, 3 for arthritis, 37 for dyslipidemia, 14 for kidney disease, 8 for asthma, 8 for digestive disease, 9 for emotional and mental disorders, 13 for liver disease, 1 for visual impairment, 1 for hearing impairment.

Table S2. Baseline characteristics of the study participants without baseline sarcopenia (n=2168)

Characteristics	Non-sarcopenia (n=1254)	Possible sarcopenia (n=806)	New-onset sarcopenia (n=108)	*P*-value
Age, year, median (IQR)	64.0 (61.0, 67.0)	65.0 (62.0, 70.0)	68.0 (64.0, 72.0)	**<0.001**
Sex (female), n (%)	537 (42.8)	416 (51.6)	50 (46.3)	**<0.001**
Married, n (%)	1076 (85.8)	625 (77.5)	82 (75.9)	**<0.001**
Residence, n (%)				**<0.001**
Urban	506 (40.4)	251 (31.1)	25 (23.2)	
Rural	748 (59.6)	555 (68.9)	83 (76.9)	
Educational level, n (%)				**<0.001**
No formal education	542 (43.2)	474 (58.8)	65 (60.2)	
Primary school	414 (33.0)	213 (26.4)	31 (28.7)	
Middle school	209 (16.7)	86 (10.7)	7 (6.5)	
High school or above	89 (7.1)	33 (4.1)	5 (4.6)	
Average household income[a], CNY, n (%)				**<0.001**
Quartile 1 (<800)	196 (21.2)	158 (25.9)	30 (34.9)	
Quartile 2 (800-3118.3)	210 (22.8)	147 (24.1)	27 (31.4)	
Quartile 3 (3118.3-8226.9)	221 (23.9)	164 (26.9)	16 (18.6)	
Quartile 4 (8226.9-95000)	296 (32.1)	141 (23.1)	13 (15.1)	
Current drinker, n (%)	458 (36.5)	231 (28.7)	32 (29.6)	**0.001**
Current smoker, n (%)	416 (33.2)	242 (30.0)	40 (37.0)	0.178

Social isolation[a], median (IQR)	1.0 (0, 1.0)	1.0 (0, 1.0)	1.0 (0, 1.0)	**<0.001**
Nighttime sleep duration[a], hours, n (%)				**<0.001**
<5.0	175 (14.0)	168 (21.1)	27 (25.5)	
5.0-6.9	453 (36.2)	251 (31.5)	34 (32.1)	
7.0-8.9	518 (41.4)	313 (39.3)	31 (29.2)	
≥9.0	104 (8.3)	64 (8.0)	14 (13.2)	
Post-lunch napping duration[a], minutes, n (%)				0.913
<30	654 (52.2)	421 (52.3)	61 (57.0)	
30-90	395 (31.5)	252 (31.3)	31 (29.0)	
≥90	203 (16.2)	132 (16.4)	15 (14.0)	
BMI (kg/m^2), median (IQR)	23.7 (21.9, 26.0)	23.7 (21.9, 25.9)	20.8 (20.1, 22.0)	**<0.001**
BMI category, n (%)				**<0.001**
Underweight	3 (0.2)	0 (0.0)	3 (2.8)	
Normal	671 (53.5)	434 (53.8)	93 (86.1)	
Overweight	434 (34.6)	265 (32.9)	5 (4.6)	
Obese	146 (11.6)	107 (13.3)	7 (6.5)	
Malnutrition[a], n (%)	85 (6.8)	52 (6.5)	7 (6.5)	0.960
CESD-10 scores, median (IQR)	6.0 (3.0, 10.0)	8.0 (4.0, 13.8)	7.0 (3.0, 12.0)	**<0.001**
Depressive symptoms, n (%)	364 (29.0)	339 (42.1)	40 (37.0)	**<0.001**
Restriction on ADL[a], n (%)[a]	149 (12.0)	159 (19.9)	21 (19.6)	**<0.001**

History of diseases, n (%)				
Hypertension[a]	409 (32.7)	319 (39.7)	24 (22.2)	**<0.001**
Diabetes[a]	94 (7.6)	78 (9.8)	5 (4.7)	0.084
Cancer[a]	10 (0.8)	3 (0.4)	3 (2.8)	**0.022**
Lung disease[a]	140 (11.2)	104 (12.9)	14 (13.0)	0.468
Heart diseases[a]	189 (15.1)	141 (17.6)	10 (9.3)	0.057
Stroke[a]	14 (1.1)	29 (3.6)	2 (1.9)	**0.001**
Arthritis[a]	409 (32.7)	318 (39.5)	39 (36.1)	**0.007**
Dyslipidemia[a]	146 (11.8)	87 (11.0)	2 (1.9)	**0.007**
Kidney disease[a]	76 (6.1)	47 (5.9)	5 (4.7)	0.831
Asthma[a]	65 (5.2)	61 (7.6)	5 (4.7)	0.070
Digestive disease[a]	271 (21.7)	178 (22.1)	27 (25.0)	0.723
Emotional and mental disorders[a]	14 (1.1)	13 (1.6)	0 (0.0)	0.301
Liver disease[a]	35 (2.8)	21 (2.6)	2 (1.9)	0.835
Comorbidity, n (%)				0.130
0	337 (26.9)	184 (22.8)	32 (29.6)	
1	377 (30.1)	241 (29.9)	35 (32.4)	
≥2	540 (43.1)	381 (47.3)	41 (38.0)	
Visual impairment[a], n (%)	82 (6.5)	73 (9.1)	10 (9.3)	0.088
Hearing impairment[a], n (%)	120 (9.6)	94 (11.7)	11 (10.2)	0.310

Cognitive scores				**<0.001**
Lowest tertile (<11)	419 (33.4)	380 (47.1)	60 (55.6)	
Middle tertile (11-14)	387 (30.9)	241 (29.9)	30 (27.8)	
Highest tertile (>14)	448 (35.7)	185 (23.0)	18 (16.7)	

Notes: Statistical significance was assessed by one-way ANOVA, Kruskal-Wallis test, or a chi-squared test.

CNY, Chinese Yuan; BMI, body mass index; CESD-10, Center for Epidemiologic Studies Depression; ADL, activities of daily living. Values of variables may not sum to 100% due to rounding. *P*-values in bold indicate <0.05.

[a]Missing data: 549 for average household income, 40 for social isolation, 16 for nighttime sleep duration, 4 for post-lunch napping duration, 2 for malnutrition, 15 for restriction on ADL, 7 for hypertension, 19 for diabetes, 11 for cancer, 6 for lung disease, 11 for heart diseases, 3 for stroke, 3 for arthritis, 36 for dyslipidemia, 12 for kidney disease, 10 for asthma, 6 for digestive disease, 8 for emotional and mental disorders, 14 for liver disease, 2 for visual impairment, 1 for hearing impairment.

Table S3. Model fit and selection criteria for LCA and LPA at baseline

	AIC	BIC	SSABIC	LRT-LMR	Entropy
LCA for sarcopenia					
2-Class	6240.026	6281.305	6259.064	<0.001	0.374
**3-Class**	**6248.026**	**6312.893**	**6277.942**	**0.5543**	**0.811**
LPA for cognition					
2-Class	26126.144	26179.217	26150.621	<0.001	0.675
**3-Class**	**25837.820**	**25920.377**	**25875.895**	**<0.001**	**0.680**
4-Class	25762.859	25874.901	25814.532	0.0003	0.673
5-Class	25760.673	25902.200	25825.944	0.1194	0.712

Notes: Better model fit is indicated by lower AIC, BIC, and SSABIC values and higher entropy values, and a non-significant value (*P*>0.05) suggests that the model with one fewer class provides a more parsimonious fit. Selected models were in bold type. AIC, Akaike Information Criteria; BIC, Bayesian Information Criteria; SSABIC, Sample-Size Adjusted Bayesian Information Criteria; LRT, *P*-value for the Lo–Mendell–Rubin Likelihood Ratio Test.

Table S4. Results of LCA and LPA with distal outcomes

A

Outcome	Latent classes [Mean score (SE)]			Overall χ^2	Pairwise comparisons χ^2		
	Non-sarcopenia (class 1)	Potential sarcopenia (class 2)	Apparent sarcopenia (class 3)		Class 1 vs. Class 2	Class 1 vs. Class 3	Class 2 vs. Class 3
Cognitive function	15.85 (0.228)	13.65 (0.132)	8.29 (0.194)	761.15***	69.89***	637.65***	523.97***

B

Outcome (Sarcopenia status)	Latent profiles [Probability (SE)]			Overall χ^2	Pairwise comparisons χ^2		
	High cognitive function (class 1)	Medium cognitive function (class 2)	Poor cognitive function (class 3)		Class 1 vs. Class 2	Class 1 vs. Class 3	Class 2 vs. Class 3
Non-sarcopenia	0.629 (0.023)	0.540 (0.022)	0.343 (0.019)				
Possible sarcopenia	0.256 (0.020)	0.309 (0.019)	0.383 (0.017)	129.43***	51.45***	106.62***	6.30*
Sarcopenia	0.115 (0.015)	0.151 (0.016)	0.274 (0.016)				

Notes: LCA for sarcopenia with continuous outcome (A) and LPA for cognition with categorical outcome (B) were run utilizing the DCON and DCAT procedures in Mplus, respectively.

*P < 0.05; ** P < 0.01; ***P < 0.001.

Table S5. Association between baseline sarcopenia and follow-up cognitive function after additional adjustment for average household income[a]

Cognitive function	Non-sarcopenia (n=1249)	Possible sarcopenia (n=752)		Sarcopenia (n=451)		β (95% CI)[c]	*P* for trend[c]
		β (95% CI)	*P*-value	β (95% CI)	*P*-value		
Model 5 (n=1791)[b]	0 (reference)	-0.63 (-1.10, -0.20)	0.004	-0.29 (-0.94, 0.36)	0.385	-0.30 (-0.59, -0.001)	0.049

Notes: [a]Linear regression analysis was used to model this association. [b]The model was performed with excluding missing data on covariates. [c]β and *P* values for trend were calculated by treating baseline sarcopenia as ordinal variables (0 = non-sarcopenia; 1 = possible sarcopenia; 2 = sarcopenia), and β represents the change in cognitive score for each one-rank increase in baseline sarcopenia status. Model 5 was adjusted for age, sex, marital status, residence, educational level, drinking status, social isolation, nighttime sleep duration, post-lunch napping duration, BMI category, malnutrition, depressive symptoms, dyslipidemia, baseline cognitive scores, and average household income.

Table S6. Association between baseline cognitive function and follow-up sarcopenia after aadditional adjustment for average household income[a]

Sarcopenia status	Lowest tertile (n=859)	Middle tertile (n=658)		Highest tertile (n=651)		OR (95% CI)[c]	*P* for trend [c]
		OR (95% CI)	*P*-value	OR (95% CI)	*P*-value		
Model 5 (n=1545)[b]	1.00 (reference)	0.91 (0.70, 1.18)	0.475	0.77 (0.57, 1.04)	0.083	0.88 (0.76, 1.02)	0.087

Notes: [a]Ordinal logistic regression was used to model this association, and the OR is interpreted as the odds of being in a more severe sarcopenia status compared with the reference category. [b]The model was performed with excluding missing data on covariates. [c]OR and *P* values for trend were calculated by treating baseline cognitive score as ordinal variables (0 = lowest tertile; 1 = middle tertile; 2 = highest tertile), and OR indicates the odds ratio of being in a more severe sarcopenia status for each one-rank increase in baseline cognitive scores (tertiles). Model 5 was adjusted for age, sex, marital status, residence, educational level, drinking status, social isolation, nighttime sleep duration, BMI category, depressive symptoms, restriction on ADL, hypertension, cancer, stroke, arthritis, dyslipidemia, baseline sarcopenia status, and average household income.

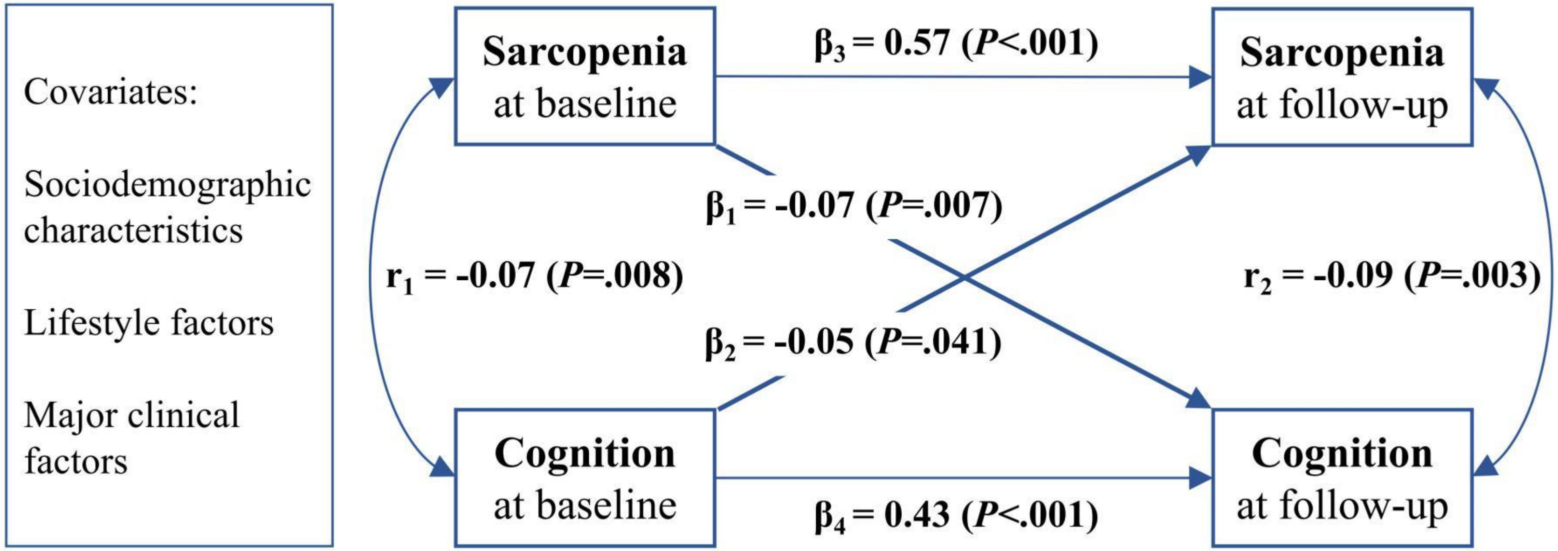

Figure S1. Cross-lagged panel model of sarcopenia and cognitive function after additional adjustment for average household income. Sarcopenia was treated as ordinal variables (0 = non-sarcopenia; 1 = possible sarcopenia; 2 = sarcopenia). The cross-lagged panel model was estimated with weighted least square mean and variance (WLSMV) that was specifically developed for ordinal variables in Mplus. Standardized coefficients were reported. Single-headed arrows represented regression paths. Double-headed arrows represented correlations.
